# Complete Genome Sequence of *Lysinibacillus sphaericus* WHO Reference Strain 2362

**DOI:** 10.1128/genomeA.00545-16

**Published:** 2016-06-09

**Authors:** Alejandra Hernández-Santana, Camilo Gómez-Garzón, Jenny Dussán

**Affiliations:** Centro de Investigaciones Microbiológicas (CIMIC), Universidad de los Andes, Bogotá, Colombia

## Abstract

*Lysinibacillus sphaericus* is a species that contains strains widely used in the biological control of mosquitoes. Here, we present the complete 4.67-Mb genome of the WHO entomopathogenic reference strain *L. sphaericus* 2362, which is probably one of the most commercialized and studied strains. Genes coding for mosquitocidal toxin proteins were detected.

## GENOME ANNOUNCEMENT

Discovered in 1965, *Lysinibacillus sphaericus*, formerly *Bacillus sphaericus*, has been increasing in importance because some members are used commercially as a biological control for the transmission of vector-borne diseases (1). *L. sphaericus* strain 2362 was isolated in 1984 from adult *Simulium damnosum* (black fly) samples in Nigeria and it was determined to belong to serotype 5a5b and phagotype 3. After its grade of mosquitocidal activity was confirmed, this strain was introduced into the WHO Collaborating Centre (Columbus, OH, USA) (2). Since then, this strain has been extensively used, not only as a reference in research about larvicidal activity of *L. sphaericus* (1, 3), but also as a point of comparison for studying metal tolerance (4, 5), the structure of surface proteins such as the S-layer (6, 7), and biosurfactant production (8).

In spite of the above, the genome of *L. sphaericus* 2362 has not been available until now, and only 13 genomes are deposited in GenBank for this bacterium. In this study, we sequenced and report the complete genome of *L. sphaericus* 2362, which is the fourth complete genome of *L. sphaericus*.

*L. sphaericus* 2362 was donated by A. Delecluse to our lab (Centro de Investigaciones Microbiológicas [CIMIC], Universidad de los Andes, Bogotá, Colombia). The genome was sequenced using Pacific Biosciences technology as a service provided by McGill University and the Génome Québec Innovation Centre (Quebec, Canada) and assembled using the Hierarchical Genome Assembly Process (HGAP) workflow (9) and Celera Assembler for the correction of sequencing errors on long reads (10). The genome was obtained as a single circular chromosomal contig with the expected size (4.67 Mb) and GC content (37.3 %), and no plasmids were detected. A total of 102,634 reads with an average length of 9,902 bp were obtained, as well as a coverage of 197×. The genome annotation was carried out using RAST (11) and the NCBI Prokaryotic Genome Annotation Pipeline (PGAP). With this analysis, 4,538 genes were found, of which 4,295 correspond to protein-coding sequences, 94 to pseudogenes, and 149 to RNA-coding genes (107 tRNAs, 37 rRNAs, and 5 ncRNAs).

In regard to larvicidal activity, binary toxin (BinA and BinB) coding genes as well as Mtx toxins (associated with the vegetative stage) were detected by tBLASTn. Interestingly, 12 copies of the S-layer gene were found. This corresponds to a previous study that reports the capability of *L. sphaericus* to regulate the S-layer expression through genomic rearrangements between multiple copies (7), although only one copy of the S-layer gene (detected by PCR) was analyzed in that study.

In summary, the complete genome of *L. sphaericus* 2362 provides a novel data set for the most studied *L. sphaericus* strain. Now, the published evidence about this bacterium could be compared with the genomic traits found herein, and future studies could be directed with a more comprehensive set of genomic evidence.

### Nucleotide sequence accession numbers.

This complete genome sequence has been deposited at DDBJ/EMBL/GenBank under the accession number CP015224. The version described in this paper is the first version, CP015224.1.
